# Tobacco quitline staffs' knowledge and attitudes about connecting quitline callers to lung cancer screening educational materials

**DOI:** 10.1002/cam4.7443

**Published:** 2024-06-28

**Authors:** Naomi Q. P. Tan, Robert J. Volk, Viola B. Leal, Jessica S. Lettieri, Linda A. Bailey, Thomas Ylioja, Paula Celestino, Lisa M. Lowenstein

**Affiliations:** ^1^ Rutgers Cancer Institute and the Robert Wood Johnson Medical School, Rutgers University New Brunswick New Jersey USA; ^2^ The University of Texas MD Anderson Cancer Center Houston Texas USA; ^3^ North American Quitline Consortium Phoenix Arizona USA; ^4^ National Jewish Health Denver Colorado USA; ^5^ Roswell Park Comprehensive Cancer Center Buffalo New York USA

**Keywords:** early detection, implementation science, lung cancer screening, lung neoplasm, shared decision‐making, tobacco cessation

## Abstract

**Objective:**

To evaluate the impact of training quitline staff in lung cancer screening (LCS) on knowledge and attitudes towards connecting quitline callers to LCS educational materials.

**Methods:**

We conducted a pre‐post evaluation within a larger implementation project in the U.S. to support LCS among quitline callers. From July 2020 to June 2021, staff from four quitline service providers completed surveys before and after training on LCS knowledge. After training, staff completed the acceptability of intervention measure, intervention appropriateness measure, and feasibility of the intervention measure.

**Results:**

A total of 245 staff completed the initial demographic survey (analytic sample), 130 completed the pre‐training survey, and 225 completed the post‐training survey. Staff were on average 47.4 years old and 76.7% were female. LCS knowledge improved after the training (*n* = 120, mean difference = +26.5%, 95% CI 21.6, 31.4, *p* < 0.001). Overall, staff felt that connecting quitline callers to LCS education materials was acceptable (*M* = 4.0, *SD* = 0.8), appropriate (*M* = 4.1, *SD* = 0.7), and feasible (*M* = 4.0, *SD* = 0.7).

**Conclusions:**

Receiving training about LCS eligibility and the benefits and harms of screening improved LCS knowledge among quitline staff. Quitline staff found that connecting callers with LCS educational materials is acceptable, appropriate, and feasible, and aligned with their primary mission.

## Graphical abstract

The objective of this study was to evaluate the impact of training quitline staff in lung cancer screening (LCS) on knowledge and attitudes towards connecting quitline callers to LCS educational materials. We found that training quitline staff on lung cancer screening significantly improved knowledge from baseline, and that quitline staff reported that educating quitline callers about LCS was acceptable, appropriate, and feasible.

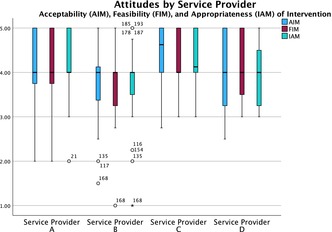

## 1 INTRODUCTION

Lung cancer is the leading cause of cancer‐related deaths in the United States,^1^ and tobacco use remains the leading risk factor for lung cancer though smoking rates have been on the decline.^2^ In 2023, an estimated 238,340 persons were diagnosed with lung cancer and 127,070 died from lung cancer.^1^ The 5‐year survival rate for non‐small cell lung cancer, the most common type of lung cancer, is 65% when diagnosed at the localized stage and 37% and 9% when diagnosed at the regional and distant stage respectively.^3^ Tobacco cessation and abstinence remain the leading strategy for reducing the burden of lung cancer; however, for people who have a heavy smoking history, the early detection of lung cancer using low‐dose computed tomography (LDCT) is also crucial for reducing lung cancer deaths and improving lung cancer survival.^4^, ^5^ Uptake and awareness of lung cancer screening (LCS) in the U.S. is still suboptimal,^6^, ^7^ and there is evidence that knowledge and awareness of LCS among eligible persons is low.^8^, ^9^ Tobacco quitlines, who provide smoking cessation services to callers interested in quitting smoking, present an opportunity to reach the screen‐eligible population to increase awareness about LCS.

A tobacco quitline consists of a partnership between two or more organizations that collaborate to fund and provide telephone‐based tobacco cessation services to tobacco users.^10^ State quitlines are funded by a state agency (most often the health department) and the services are provided by a call center with special expertise in tobacco cessation.^11^ There are 53 publicly funded quitlines in the U.S., including 50 states, the District of Columbia, Puerto Rico and Guam.^11^ The types of services provided by quitlines, number of people served, reporting requirements, and payment levels are specified by the state agency in the contract. Although the state relies on the call center as an expert on tobacco cessation services and often engages in consensus decision making regarding the types of services to provide, the final decision on services is determined by the state agency that funds the quitline. In the U.S., 11 quitline service providers operate the 53 state quitlines.^12^ Quitlines have been successfully operated in the U.S. as they are convenient and accessible to most people by minimizing common barriers to accessing health services, such as transportation or lack of time.^10^, ^13^ Many studies have found that telephone counseling increased the quit success rate of people who smoke, particularly when multiple counseling sessions are conducted.^14^, ^15^, ^16^ In addition, given that tobacco quitlines have been steadily increasing their use of web‐based services or interventions,^17^ we expect that their ability to reach potentially LCS‐eligible callers will only widen.

The North American Quitline Consortium (NAQC) estimates that more than half of all U.S. quitline callers (200,000 persons) would be eligible for LCS based on age and smoking history.^18^, ^19^ There is a unique opportunity to leverage tobacco quitlines by expanding their mission to include identification and support for callers who meet eligibility for LCS and might benefit from LCS. In this project, we trained quitline call center staff from two multistate and two single‐state service providers to identify callers who met eligibility for LCS, based on age and smoking history, and facilitate access to resources that support LCS. Here we report on the training strategies, impact of the training on staff members' knowledge of LCS, and evaluate the implementation potential (acceptability, appropriateness, and feasibility) of connecting quitline callers with decision support materials about LCS.

## 2 MATERIALS AND METHODS

### 2.1 Project CONNECT overview

Project CONNECT was a PCORI‐funded project where quitline staff were trained to identify quitline callers that were potentially eligible for LCS and to connect them with decision support materials about LCS. Quitline staff did not deliver the counseling and SDM visit for LCS to callers. Four service providers representing seven states participated in Project CONNECT. All service providers provide telephone‐based tobacco treatment services to callers; Service Provider A is affiliated with a respiratory care hospital and operates over 20 state quitlines, Service Provider B is a healthcare service provider that operates over 20 state quitlines, Service Provider C is a voluntary health organization, and Service Provider D is affiliated with a comprehensive cancer center. Quitline staff completed a demographic survey, pre‐training survey, attended a 60‐min training about lung cancer and screening (either a live or recorded webinar), and completed a post‐training survey 1 month after the training. The study was approved by our Institutional Review Board (protocol number blinded).

### 2.2 Procedure

Participants included intake staff, tobacco treatment specialists, and/or managers. Invitations to the project were distributed by service providers. Quitline staff received a recruitment email describing the study. The email included a link to the informed consent, demographic survey, and pre‐training survey (containing the knowledge items). After completing the pre‐training survey, quitline staff were asked to complete a 60‐min training about LCS. The webinar content was adapted from the Agency for Healthcare Research and Quality (AHRQ) SHARE program on LCS. The training was delivered as a narrated webinar that covered lung cancer facts, the risks and benefits of LCS, use of LDCT, LCS eligibility, and LCS educational resources available at *lungscreen.health*. Staff completed the training by attending the live webinar or watching a recorded video. Three service providers had the research team deliver the training, and one service provider used the train‐the‐trainer approach. Quitline staff could complete the training without participating in the pre‐training or post‐training survey. The post‐training survey was delivered 1 month after the training and assessed knowledge and attitudes towards connecting quitline callers to LCS educational materials. If a staff member did not complete the pre‐training survey, they were sent the consent, demographic survey, and post‐training survey after they had completed training.

### 2.3 Measures

#### 2.3.1 Knowledge

We used a previously published and validated measure of LCS knowledge developed by the research team.^20^ The 11 items assessed knowledge about LCS eligibility, potential benefits, and potential harms. All knowledge items were close‐ended questions. Sample knowledge items include (1) “Do health professional groups recommend all current and former smokers be screened for lung cancer?” (Response options: Yes, No, Unsure) and (2) “Without screening, is lung cancer often found at a later stage when cure is less likely?” (Response options: Yes, No, Unsure). Response options were recoded as Correct (scored as 1) or Incorrect (includes ‘Unsure’ responses) (scored as 0). Participants' recoded responses on the knowledge items were summed to form a total LCS knowledge score (range of 0–11) and then standardized as a percentage of correct responses on the LCS knowledge scale.

#### 2.3.2 Attitudes

To evaluate quitline staff's attitudes towards connecting quitline callers to educational materials on LCS, we used three validated measures of implementation attitudes, including: (1) Acceptability of Intervention Measure (AIM; four items), for example, “The process of identifying potentially‐eligible callers and referring them to the LCS website meets my approval”; (2) Feasibility of Intervention Measure (FIM; four items), for example, “The process of identifying potentially‐eligible callers and referring them to the LCS website seems implementable”; and (3) Intervention Appropriateness Measure (IAM; four items), for example, “The process of identifying potentially eligible callers and referring them to the LCS website seems fitting.”^21^ The responses were measured on a 5‐point Likert scale from Strongly Disagree to Strongly Agree. The means of the four items of each measure were used in the analyses.

#### 2.3.3 Demographics

The survey asked quitline staff their age, gender, race, ethnicity, education level, tenure at the quitline, and role at the quitline.

### 2.4 Statistical analysis

We conducted descriptive analysis for participant characteristics, compared mean pre‐ and post‐scores for each question and overall mean pre‐training and post‐training scores with a paired *t*‐test. Mean difference scores were only calculated for three of the quitline service providers, as quitline staff at one quitline service provider completed only the post‐training survey (except for one quitline staff who completed the pre‐training survey). For LCS knowledge, we defined being well‐informed as having 80% correct responses.

To compare differences in post‐training knowledge among service providers, we used a one‐way analysis of covariance with race as a covariate. To examine differences in attitudes between service providers, we used a one‐way analysis of covariance with race as a covariate. All statistical tests were two‐sided with a significance level of 0.05. Statistical analysis was performed with Stata™. To examine differences in attitudes between service providers, we used a one‐way analysis of covariance with race as a covariate. All statistical tests were two‐sided with a significance level of 0.05. Statistical analysis was performed with Stata™.

## 3 RESULTS

### 3.1 Participant characteristics

**FIGURE 1:**
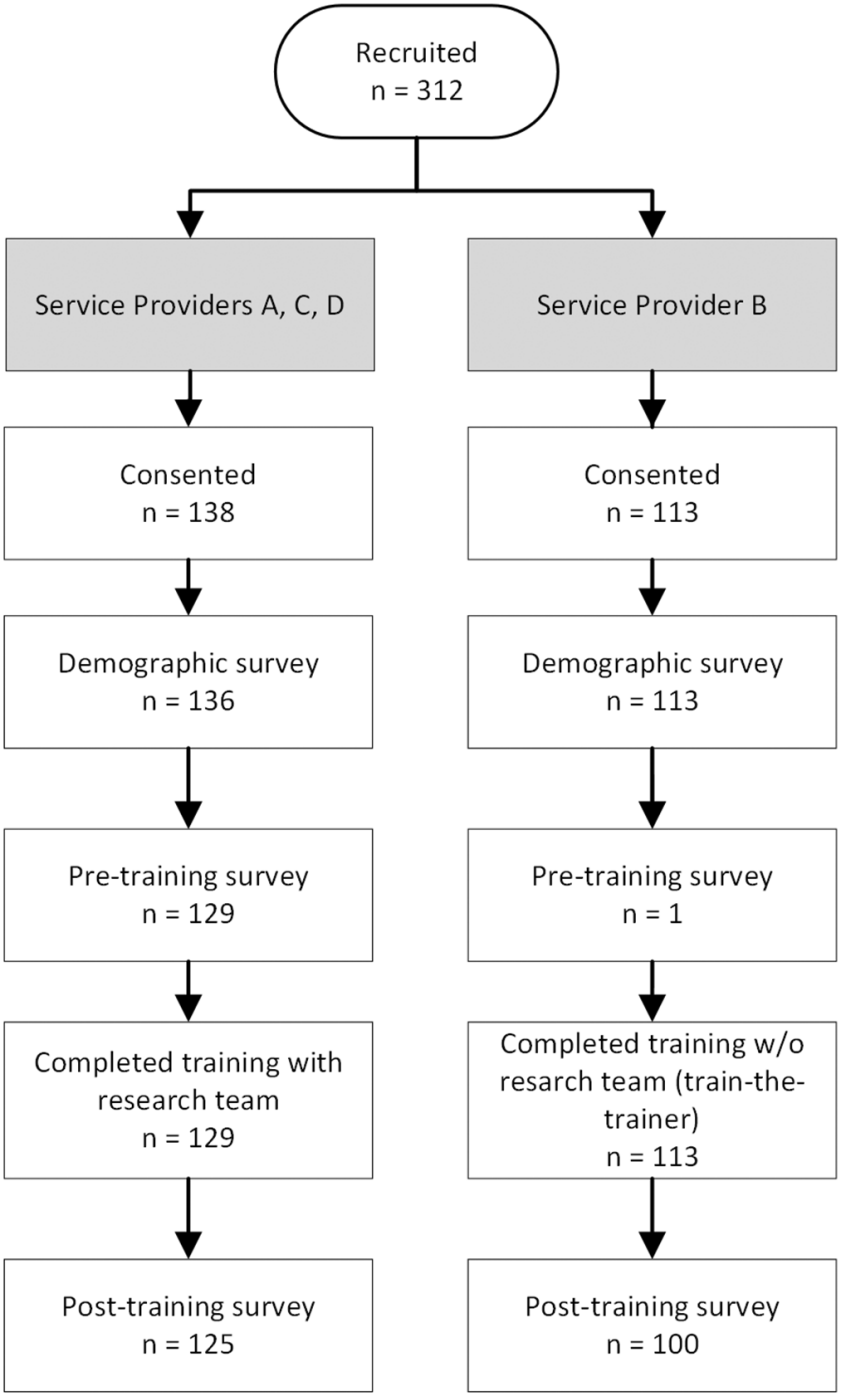
Consort Diagram.The analytic sample consisted of all participants who consented to the study and completed the demographic survey (*N* = 245). Participants were allowed to complete subsequent surveys if they had not completed previous surveys or attended training. Participants at Service Provider B elected to use the train‐the‐trainer approach instead of having the research team deliver the training. Hence, participants at Service Provider B did not complete the pre‐training survey (except for 1 participant).

Of the 312 staff members invited to the study, a total of 245 consented and contributed demographic data, forming our analytic sample (Figure 1). At Service Provider A, C, and D, 136 quitline staff completed the demographic survey, 129 completed the pre‐training survey, 129 completed training with the research team, and 125 completed the post‐training survey. At Service Provider B, 109 quitline staff completed the demographic survey, only one completed the pre‐training survey, 113 underwent LCS training using the train‐the‐trainer approach, and 100 completed the post‐training survey. Two staff members left the organization after completing the pre‐training survey (did not complete training) and three staff members left their organization after completing training (did not complete the post‐training survey). The participants had a mean age of 47.4 years (*SD* = 12.2), 76.7% (188/245) were female, 52.2% (128/245) were White, 75.1% (184/245) were not Hispanic or Latino, and 73.9% (181/245) had a college degree or higher. Table 1 shows participant characteristics by quitline service provider, and Table A1 shows participant characteristics by survey completion status (e.g., demographics only, pre‐training only, post‐training only, and pre‐ and post‐training).

**TABLE 1:** Participant characteristics by service provider (*N* = 245^a^).

	Service provider				
	(A). *n* = 84	(B). *n* = 109	(C). *n* = 30	(D). *n* = 22	
	*n* (%)	*n* (%)	*n* (%)	*n* (%)	*p* ^b^
Age	Mean = 44.8 (*SD* = 13.4) Range [22–72]	Mean = 47.9 (*SD* = 10.8) Range [27–75]	Mean = 49.6 (*SD* = 12.0) Range [27–70]	Mean = 51.5 (*SD* = 12.5) Range [29–71]	0.07
Gender	0.28				
Female	61 (72.6)	82 (75.2)	25 (83.3)	20 (90.9)	
Male	22 (26.2)	24 (22.0)	5 (16.7)	1 (4.5)	
Other	1 (1.2)	‐	‐	‐	
Prefer not to answer	‐	3 (2.8)	‐	1 (4.5)	
Race					0.75
American Indian or Alaska Native	3 (3.6)	4 (3.7)	‐	‐	
Asian	3 (3.6)	4 (3.7)	1 (3.3)	‐	
Black or African American	12 (14.3)	20 (18.3)	4 (13.3)	4 (18.2)	
Native Hawaiian or Pacific Islanders	‐	‐	‐	‐	
White	43 (51.2)	53 (48.6)	21 (70.0)	11 (50.0)	
Two or more races	14 (16.7)	16 (14.7)	3 (10.0)	2 (9.1)	
Prefer not to answer	9 (10.7)	12 (11.0)	1 (3.3)	5 (22.7)	
Ethnicity					< 0.01
Hispanic or Latino	16 (19.0)	20 (18.3)	2 (6.7)	1 (4.6)	
Not Hispanic or Latino	63 (75.0)	80 (73.4)	27 (90.0)	14 (63.6)	
Prefer not to answer	5 (6.0)	9 (8.3)	1 (3.3)	7 (31.8)	
Education					0.18
Graduated high school /GED	3 (3.6)	1 (0.9)	2 (6.7)	1 (4.5)	
Some college/Trade school	11 (13.1)	28 (25.7)	6 (20.0)	5 (22.7)	
Graduated college	47 (56.0)	55 (50.5)	19 (63.3)	10 (45.5)	
Graduate degree	22 (26.2)	21 (19.3)	3 (10.0)	4 (18.2)	
Prefer not to answer	1 (1.2)	4 (3.5)	‐	2 (9.1)	

^a^ The full analytic sample (*N* = 245) included all participants who completed the demographic survey.

^b^ Differences in mean age by service provider were examined using a one‐way analysis of variance and differences in gender, race, ethnicity, and education by service provider were examined using chi‐square tests.

### 3.2 LCS knowledge

Table 2 shows the percentage correct knowledge scores at pre‐training and post‐training across service providers. Across service providers, 130 quitline staff completed the pre‐training survey. At pre‐training, the percentage of total correct responses for Service Provider A, C, and D was 29.0%, 44.2%, and 47.7% respectively. The pre‐training score differed significantly between quitline service providers (*F* [3,126] = 7.2, *p* < 0.001). Compared to Service Provider A, Service Provider C and D had significantly higher pre‐training scores. There was no significant difference in pre‐training scores between Service Provider C and D. There were no differences in pre‐training knowledge between gender (male vs. female), race (White vs. non‐White), ethnicity (Hispanic vs. non‐Hispanics), and education (at least some college vs. no college) (Table A2).

**TABLE 2:** Percentage correct knowledge scores at pre‐training and post‐training surveys by service provider (n = 225)^a^.

	Pre‐training Knowledge *n* = 130	Post‐training Knowledge *n* = 225^b^	Mean difference *n* = 120^c^
	% (*SD)*	% (*SD*)	% (*SD*)
Total	35.0 (22.1)	53.7 (26.7)	26.5 (27.0)
Service Provider A	29.0 (18.4)	54.7 (26.6)	26.2 (28.1)
Service Provider B^d^	‐	44.5 (23.7)	‐
Service Provider C	44.2 (25.9)	73.1 (22.2)	28.6 (23.3)
Service Provider D	47.7 (21.2)	68.0 (26.3)	24.9 (29.0)
	*F*(3,126) = 7.2, *p* < 0.001, $\eta^{2}$ = 0.15	*F*(3,220) = 11.9, *p* < 0.001, $\eta^{2}$ = 0.14	*F*(3,116) = 0.1, *p* = 0.97, $\eta^{2}$ = 0.002

^a^ The sample size of 225 includes participants who completed the pre‐ and/or post‐training survey.

^b^ Differences in post‐training knowledge among service providers was examined using a one‐way analysis of covariance controlling for race (White vs. non‐White).

^c^ The mean difference in knowledge scores was only calculated for participants who completed both the pre‐ and post‐training surveys (*n* = 120).

^d^ Only 1 quitline staff at Service Provider B contributed data in the pre‐training survey (knowledge score of 9.1%) while the rest did not complete the pre‐training survey. The pre‐training knowledge mean and mean difference in pre‐ and post‐training knowledge were not calculated.

Across service providers, 225 quitline staff members completed the post‐training survey. At post‐training, the percentage of total correct responses for Service Provider A, B, C, and D was 54.7%, 44.5%, 73.1%, and 68.0% respectively. None of the service providers had post‐training knowledge scores exceeding 80% correct responses. Similarly, controlling for race, the post‐training score differed significantly between quitline service providers (*F* [3,220] = 11.9, *p* < 0.001). Service Provider C had higher post‐training scores than both Service Provider A and B. Service Provider A and D both had higher post‐training scores compared to Service Provider B. There were no differences in post‐training knowledge between gender (male vs. female), ethnicity (Hispanic vs. non‐Hispanics), and education (at least some college vs. no college) (Table A2).

We examined the difference in LCS knowledge among quitline staff members at the three quitline service providers (A, C, and D) that completed both pre‐ and post‐training surveys (*n* = 120; Table 2). Across the three quitline service providers, after the training, LCS knowledge significantly increased (Mean difference = 26.5%, *SD* = 27.0, 95% CI: 21.6, 31.4). There was also no statistically significant difference in change in LCS knowledge after training between the quitline service providers.

### 3.3 Acceptability, appropriateness, feasibility of disseminating educational materials on LCS

Overall, staff across all quitline service providers felt that incorporating LCS education was acceptable (AIM: Mean = 4.0 out of 5, *SD* = 0.8), feasible (FIM: Mean = 4.0 out of 5, *SD* = 0.7), and appropriate (IAM: Mean = 4.1 out of 5, *SD* = 0.7). Although we found significant differences in race, White compared to non‐White staff, we found no differences for other demographic variables (Table A2).

**FIGURE 2:**
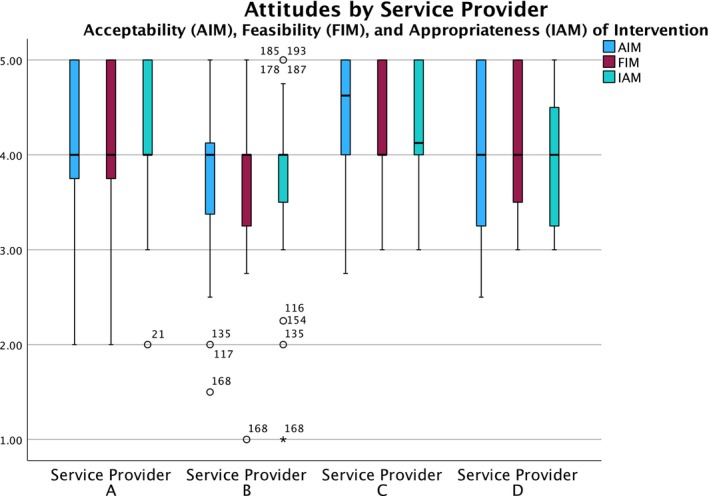
Differences in attitudes towards dissemination of lung cancer screening educational materials (Acceptability of Intervention Measure; AIM), feasibility (Feasibility of Intervention Measure; FIM), and appropriateness (Intervention Appropriateness Measure; IAM) by service provider.

For AIM, Service Provider A (Mean difference = 0.3, *SE* = 0.1, 95% CI: 0.1, 0.5) and Service Provider C (Mean difference = 0.5, *SE* = 0.2, 95% CI: 0.2, 0.8) both reported higher acceptability compared to Service Provider B. For FIM, Service Provider A (Mean difference = 0.3, *SE* = 0.1, 95% CI: 0.1, 0.5) reported higher feasibility compared to Service Provider B. For IAM, Service Provider A (Mean difference = 0.3, *SE* = 0.1, 95% CI: 0.1, 0.5) and Service Provider C (Mean difference = 0.3, *SE* = 0.2, 95% CI: 0.03, 0.6) both reported higher acceptability compared to Service Provider B. Figure 2 summarizes attitudes towards disseminating LCS education materials among service providers.

Differences between service providers remained after controlling for race (Table 3). White quitline staff had higher acceptability (Mean = 4.2, *SD* = 0.8) than non‐White staff (Mean = 3.8, *SD* = 0.7) (*d* = 0.4, 95% CI: 0.2, 0.7). White quitline staff reported higher perceived feasibility (Mean = 4.1, *S*D = 0.7) than non‐White staff (Mean = 3.9, *SD* = 0.7) (*d* = 0.3, 95% CI: 0.03, 0.6). White quitline staff reported higher perceived appropriateness (Mean = 4.2, *SD* = 0.8) than non‐White staff (Mean = 3.9, *SD* = 0.7) (*d* = 0.4, 95% CI: 0.1, 0.7).

**TABLE 3:** Attitudes towards dissemination of lung cancer screening educational materials by service provider (*n* = 225)^a^.

	Acceptability of Intervention (AIM)	Feasibility of Intervention (FIM)	Appropriateness of Intervention (IAM)
	Mean^b^ (*SD*)	Mean (*SD*)	Mean (*SD*)
All	4.0 (0.8)	4.0 (0.7)	4.1 (0.7)
Service Provider A	4.1 (0.7)	4.2 (0.7)	4.2 (0.7)
Service Provider B	3.9 (0.7)	3.9 (0.7)	3.9 (0.8)
Service Provider C	4.3 (0.7)	4.3 (0.7)	4.2 (0.7)
Service Provider D	4.0 (0.9)	4.1 (0.7)	4.0 (0.7)
	*F* (3, 220) = 4.3, *p* < 0.01, $\eta^{2}$ = 0.6	*F* (3,220) = 4.2, *p* < 0.01, $\eta^{2}$ = 0.6	*F* (3,220) = 2.9, *p* < 0.05, $\eta^{2}$ = 0.4

^a^ The sample size of 225 includes participants who completed the post‐training survey, where AIM, FIM, and IAM measures were asked. Differences in attitudes among service providers were examined using an analysis of covariance controlling for race (White vs. non‐White).

^b^ AIM, FIM, and IAM scores range from 0 to 5.

## 4 DISCUSSION

Tobacco quitlines can play a critical role in connecting quitline callers, many of whom are eligible for LCS based upon age and smoking history, to high‐quality decision support materials on LCS.^22^ We found that the training session significantly improved quitline staffs' knowledge of LCS from baseline, and that quitline staff thought that connecting quitline callers to LCS education was acceptable, feasible, and appropriate.

Our findings show that overall, quitline staff had low levels of baseline knowledge of LCS, indicating that there is a need for training about lung cancer and LCS so they can educate quitline callers, particularly about the eligibility criteria and the potential benefits and harms of LCS. None of the service providers had post‐training knowledge scores exceeding 80% correct responses, indicating that more intensive LCS education may be required for quitline staff to meet our threshold for being well‐informed. This is especially important if quitline staff were educating callers on LCS, but our study had quitline staff connect callers with educational materials. While LCS knowledge significantly improved after the training, the differences between service providers were mainly driven by Service Provider B, whose staff elected to use the train‐the‐trainer model. Service Provider B's post‐training knowledge scores were similar to pre‐training scores from other service providers that had their staff attend live webinar trainings delivered by the research team or watch a recorded video. As majority of Service Provider B staff did not complete the pre‐training survey, we were unable to fully ascertain if the knowledge increases were a result of the training. However, the relatively lower post‐training knowledge scores in Service Provider B compared to other service providers may suggest that having training delivered by the research team may be more a more effective approach to increase knowledge about LCS. This may be due to reasons such as fidelity to the training materials, how information in the training materials is communicated to the staff and having a research team member with expertise in LCS available to answer questions during the training. Although the train‐the‐trainer approach is a more sustainable and cost‐effective education strategy, there is currently insufficient evidence that knowledge and skills are effectively transferred from master trainer to trainers in the health domain.^23^ Hence, we recommend that training be delivered by a content expert as sustainable education methods continue to be evaluated. In addition, future studies may also consider additional post‐training surveys beyond 1 month of the training to assess declines in knowledge over time which can inform the length of interval between retraining to maintain knowledge levels.

Previous efforts, such as the Smoking Cessation at Lung Examination (SCALE) Collaboration, have focused on integrating smoking cessation treatment in LCS programs.^24^, ^25^ However, few studies have examined how LCS can be integrated into smoking cessation efforts by tobacco quitline service providers.^26^, ^27^, ^28^ Promoting LCS through quitlines is a strategy that combines primary prevention (cigarette use cessation) with a proven secondary prevention strategy (screening with LDCT). Little is known about how quitline staff perceive connecting quitline callers with information about LCS.

Our study found that quitline staff thought that connecting callers to these LCS materials was acceptable, feasible, and appropriate. Quitline staff reported that they liked and approved of this intervention and felt that the intervention was a good match with the mission of the tobacco quitlines and their role as a quitline staff member. In addition, quitline staff also thought that it was implementable and doable to connect quitline callers with LCS resources. Although attitudes towards disseminating LCS educational materials was slightly lower at Service Provider B, the scores still indicated high acceptability, feasibility, and appropriateness. Similar to knowledge scores, the lower attitudes scores may have been due to the use of the train‐the‐trainer approach. For instance, a research team member may be able to communicate the importance of the intervention more effectively or be able to adequately address implementation questions or concerns from quitline staff. Nevertheless, these findings indicate support among quitline staff for LCS in the tobacco treatment context, which is important for future dissemination and implementation of this intervention to other tobacco quitlines. Our findings are also consistent with a previous study that found that tobacco treatment specialists lack knowledge about LCS but feel that it is important for their callers.^26^ However, the study's sample of tobacco treatment specialists worked mainly in clinical settings, while our study focused on quitline staff.^27^ Given the acceptability of connecting quitline callers with educational materials about LCS, an important next step would be to further leverage ongoing efforts by tobacco quitline service providers by training quitline staff to deliver LCS education to callers. Quitline staff can be trained to conduct shared decision‐making for LCS, in line with the Centers for Medicare and Medicaid's (CMS) requirements, to ensure that callers are informed about the benefits and harms of LCS and have their preferences assessed prior before making a decision about screening.

This project has several limitations. First, as mentioned previously, quitline staff at Service Provider B did not complete the pre‐training survey, which limited comparisons of knowledge scores from before and after the training for nearly half the sample. However, we had pre and posttest knowledge data from the three other service providers. Given that this was an implementation project and not a research study, we adapted delivery of the training to the preferences of the service providers to engage them in meaningful project collaborations. Second, we did not have a control group that did not receive LCS training, which limited our analysis to knowledge changes from before to after the training. Finally, our study included quitline staff from four quitline service providers only. However, these four quitline service providers collectively cover about 85% of the U.S. state or territories that have quitlines. Third, recall of the pre‐training knowledge questions may have contributed to performance in the post‐training knowledge questions, although participants were not given the correct answers to the knowledge questions at any point. For individual service providers, the median time between training and completion of the post‐training survey was 22 days for Service Provider A, 14 days for Service Provider B, 2 days for Service Provider C, and 1 day for Service Provider D. Two participants were outliers and completed the survey more than 60 days after the training and were not included in the median. There was a quick turnaround for Service Provider C and D, which may contribute to better performance on the knowledge questions. For Service Provider A, 22 days may have been sufficient to minimize memory effects. Service Provider B participants did not complete the pre‐training survey.

Quitline callers are an important and underappreciated population for LCS efforts. A large number of callers will be eligible for screening based on their smoking history and age, and callers calling in for smoking cessation treatment are already likely to be motivated to improve their health. Hence, quitline staff may play a crucial role in educating callers, increasing LCS awareness, and potentially improving LCS uptake and decisions. As we move towards translating evidence‐based interventions into routine practice by educating quitline service deliverers on LCS content, we have found that who conducts the training has bearing on learning of the content. Research team members who are the content experts are well‐placed to conduct the training and ensure that quitline staff have the knowledge to educate callers on LCS. Finally, funding for tobacco quitlines is highly variable across states and the services provided by quitlines are driven by funding availability.^29^, ^30^ It is reassuring that our study findings show that quitline staff find that connecting callers with LCS educational materials was aligned with their primary mission, indicating that this would not detract from their main services.

## AUTHOR CONTRIBUTIONS

**Naomi Q. P. Tan:** Formal analysis (equal); visualization (equal); writing – review and editing (equal). **Robert J. Volk:** Conceptualization (lead); data curation (lead); funding acquisition (lead); investigation (equal); methodology (equal); supervision (equal); writing – review and editing (equal). **Viola B. Leal:** Funding acquisition (supporting); project administration (supporting); supervision (supporting); writing – review and editing (equal). **Jessica S. Lettieri:** Data curation (supporting); investigation (supporting); project administration (supporting); supervision (supporting); writing – original draft (supporting); writing – review and editing (supporting). **Linda A. Bailey:** Conceptualization (equal); funding acquisition (equal); investigation (equal); writing – review and editing (equal). **Thomas Ylioja:** Conceptualization (equal); funding acquisition (supporting); investigation (equal); resources (equal); writing – review and editing (equal). **Paula Celestino:** Funding acquisition (supporting); investigation (equal); resources (equal); writing – review and editing (equal). **Lisa M. Lowenstein:** Conceptualization (equal); formal analysis (lead); funding acquisition (lead); investigation (lead); methodology (lead); project administration (lead); supervision (equal); writing – original draft (lead); writing – review and editing (lead).

## FUNDING INFORMATION

Research reported in this work was funded through a Patient‐Centered Outcomes Research Institute (PCORI) Award (DI‐2018C3‐14825) and a grant from NIH/NCI under award number P30CA016672 and used the Decision Science Core and Clinical Protocol and Data Management System. The views, in this work are solely the responsibility of the authors and do not necessarily represent the views of the Patient‐Centered Outcomes Research Institute (PCORI), its Board of Governors or Methodology Committee.

## CONFLICT OF INTEREST STATEMENT

Ms. Bailey reports that NAQC receives membership fees from quitlines. Other authors have no conflicts of interest to disclose.

## ETHICS STATEMENT

This study was approved by The University of Texas MD Anderson Cancer Center's Institutional Review Board.

## Data Availability

The data that support the findings of this study are available from the corresponding author upon reasonable request.

## APPENDIX A

**TABLE A1:** Participant characteristics by survey completion status.

	Demographic survey only (*n* = 10)	Pre‐training survey only (*n* = 10)	Post‐training survey only (*n* = 105)	Pre‐ and post‐training surveys (*n* = 120)	
	*n* (%)	*n* (%)	*n* (%)	*n* (%)	*p* ^a^
Age	Mean = 40.7 (*SD* = 9.1) Range [30–62]	Mean = 44.2 (*SD* = 11.8) Range [28–61]	Mean = 48.8 (*SD* = 10.8) Range [27–75]	Mean = 47.0 (*SD* = 13.4) Range [22–72]	0.16
Gender					< 0.01
Female	8 (80.0)	6 (60.0)	78 (74.3)	96 (80.0)	
Male	2 (20.0)	3 (30.0)	24 (22.9)	23 (19.2)	
Other	‐	1 (10.0)	‐	‐	
Prefer not to answer	‐	‐	3 (2.9)	1 (0.8)	
Race					0.15
American Indian or Alaska Native	1 (10.0)	‐	4 (3.8)	2 (1.7)	
Asian	2 (20.0)	‐	3 (2.9)	3 (2.5)	
Black or African American	1 (10.0)	1 (10.0)	20 (19.0)	18 (15.0)	
Native Hawaiian or Pacific Islanders	‐	‐	‐	‐	
White	2 (20.0)	4 (40.0)	54 (51.4)	68 (56.7)	
Two or more races	3 (30.0)	3 (30.0)	13 (12.4)	16 (13.3)	
Prefer not to answer	1 (10.0)	2 (20.0)	11 (10.5)	13 (10.8)	
Ethnicity					0.21
Hispanic or Latino	1 (10.0)	1 (10.0)	20 (19.0)	17 (14.2)	
Not Hispanic or Latino	9 (90.0)	6 (60.0)	75 (71.4)	94 (78.3)	
Prefer not to answer	‐	3 (30.0)	10 (9.5)	9 (7.5)	
Education					0.21
Graduated high school /GED	‐	‐	1 (1.0)	6 (5.0)	
Some college/Trade school	3 (30.0)	1 (10.0)	26 (24.8)	20 (16.7)	
Graduated college	5 (50.0)	3 (30.0)	54 (51.4)	69 (57.5)	
Graduate degree	2 (20.0)	5 (50.0)	20 (19.0)	23 (19.2)	
Prefer not to answer	‐	1 (10.0)	4 (3.8)	2 (1.7)	

^a^ Differences in mean age by completion status were examined using a one‐way analysis of variance and differences in race, ethnicity, and education by completion status were examined using chi‐square tests.

**TABLE A2:** Demographic differences in knowledge and attitudes for covariate analysis.^a^

	Pre‐training knowledge	Post‐training knowledge	Acceptability of intervention (AIM)	Feasibility of intervention (FIM)	Appropriateness of intervention (IAM)
	Mean % (*SD*)	Mean % (*SD*)	Mean (*SD*)	Mean (*SD*)	Mean (*SD*)
Gender					
Male	33.8 (21.4)	56.3 (24.6)	4.1 (0.8)	4.1 (0.8)	4.1 (0.8)
Female	35.3 (22.4)	52.9 (27.2)	4.0 (0.8)	4.0 (0.7)	4.0 (0.7)
	Mean diff = −2.2, 95% CI: −11.1, 6.7	Mean diff = 4.2, 95% CI: −4.1, 12.6	Mean diff = 0.1, 95% CI: −0.2, 0.3	Mean diff = 0.1, 95% CI: −0.2, 0.3	Mean diff = 0.03, 95% CI: −0.2, 0.3
Race					
White	38.0 (23.1)	58.5 (26.8)	4.2 (0.8)	4.1 (0.7)	4.2 (0.8)
Non‐White	31.2 (20.3)	47.9 (25.5)	3.8 (0.7)	3.9 (0.7)	3.9 (0.7)
	Mean diff = 5.6, 95% CI: −1.6, 12.8	Mean diff = 10.2, 95% CI: 3.3, 17.1	Mean diff = 0.3, 95% CI: 0.1, 0.5	Mean diff = 0.2, 95% CI: 0.03, 0.4	Mean diff = 0.3, 95% CI: 0.1, 0.5
Ethnicity					
Hispanic	27.3 (17.4)	47.4 (24.3)	3.8 (0.7)	3.9 (0.6)	3.9 (0.7)
Non‐Hispanic	36.2 (22.6)	54.9 (27.0)	4.1 (0.8)	4.1 (0.7)	4.1 (0.8)
	Mean diff = −5.1, 95% CI: −15.6, 5.5	Mean diff = −6.3, 95% CI: −15.8, 3.2	Mean diff = −0.2, 95% CI: −0.5, 0.04	Mean diff = −0.2, 95% CI: −0.4, 0.1	Mean diff = −0.3, 95% CI: −0.5, 0.004
Education level					
At least some college	35.2 (21.7)	53.7 (26.3)	4.0 (0.8)	4.0 (0.7)	4.1 (0.8)
No college	32.3 (28.1)	53.2 (32.7)	3.9 (0.8)	3.9 (0.6)	3.8 (0.6)
	Mean diff = 6.6, 95% CI: −7.6, 20.9	Mean diff = 1.1, 95% CI: −13.9, 16.1	Mean diff = 0.2, 95% CI: −0.3, 0.6	Mean diff = 0.2, 95% CI: −0.2, 0.6	Mean diff = 0.3, 95% CI: −0.2, 0.7

^a^ Mean differences in knowledge and attitudes by demographic variables were calculated using an analysis of covariance controlling for service provider, as there are significant differences in knowledge and attitudes by service provider.
